# Genome-Wide Association Study for Muscle Fat Content and Abdominal Fat Traits in Common Carp (*Cyprinus carpio*)

**DOI:** 10.1371/journal.pone.0169127

**Published:** 2016-12-28

**Authors:** Xianhu Zheng, Youyi Kuang, Weihua Lv, Dingchen Cao, Zhipeng Sun, Xiaowen Sun

**Affiliations:** 1 National & Local United Engineering Laboratory of Freshwater Fish Breeding, Heilongjiang River Fisheries Research Institute, Chinese Academy of Fishery Sciences, Harbin, Heilongjiang, China; 2 College of Life Science, Northeast Agricultural University, Harbin, Heilongjiang, China; Xiamen University, CHINA

## Abstract

Muscle fat content is an important phenotypic trait in fish, as it affects the nutritional, technical and sensory qualities of flesh. To identify loci and candidate genes associated with muscle fat content and abdominal fat traits, we performed a genome-wide association study (GWAS) using the common carp 250 K SNP assay in a common carp F_2_ resource population. A total of 18 loci surpassing the genome-wide suggestive significance level were detected for 4 traits: fat content in dorsal muscle (MFdo), fat content in abdominal muscle (MFab), abdominal fat weight (AbFW), and AbFW as a percentage of eviscerated weight (AbFP). Among them, one SNP (carp089419) affecting both AbFW and AbFP reached the genome-wide significance level. Ten of those loci were harbored in or near known genes. Furthermore, relative expressions of 5 genes related to MFdo were compared using dorsal muscle samples with high and low phenotypic values. The results showed that 4 genes were differentially expressed between the high and low phenotypic groups. These genes are, therefore, prospective candidate genes for muscle fat content: ankyrin repeat domain 10a (*ankrd10a*), tetratricopeptide repeat, ankyrin repeat and coiled-coil containing 2 (*tanc2*), and four jointed box 1 (*fjx1*) and choline kinase alpha (*chka*). These results offer valuable insights into the complex genetic basis of fat metabolism and deposition.

## Introduction

Common carp (*Cyprinus carpio*) is one of the most widely cultured fish species in the world. In 2012, the world production of common carp exceeded 4.16 million tons and, in China, the yield reached more than 3.17 million tons (ftp://ftp.fao.org/FI/STAT/summary/default.htm, archived on July 18, 2016). The species is important in the global aquaculture industry, and it is also a traditional food in China. In addition, it serves as a model species for many research areas, e.g. toxicology, ecology, physiology, nutrition and evolution. Significant progress has been made in developing common carp genomic tools in recent years. A greater amount of genomic resources is available for common carp research, such as a medium-density [1] and three high-density [2–4] genetic linkage maps, BAC libraries [5,6] and physical maps [7], and high-throughput genotyping chips [8]. Furthermore, the common carp genome has been sequenced and assembled [9], which provides important reference for genomic and comparative genomic researches of Cyprinidae fish and other species. All of these resources are available on www.carpbase.org, laying the foundation for future studies on the genetic architectures of economic traits in common carp and related species.

Genetic improvement of the flesh quality traits has become a great concern to the aquaculture industry, as the aquatic products must meet the requirements for safety, nutrition and quality[10]. For flesh quality traits like fat content, fatty acid, fillet color, drop loss, texture, and other fat traits such as the contents and proportions of abdominal fat, the traditional selection is not possible as it is impossible measurements on living animals. However, thanks to the rapid development of marker/gene-assisted selection techniques, it is now easy and convenient to study on these traits. Therefore, identification of molecular markers associated with these traits is an necessary step towards genetic improvement of these traits [11].

Muscle fat content affects the organoleptic quality, texture, flavor and nutritional value of flesh and thus it is the most important attribute of flesh quality [12]. So far, progress has been made in studies on genetic basis of muscle fat content trait in a few cultured fish species. The heritability estimates for fat content in common carp was 0.58 [13]. In Atlantic salmon, the heritability for fat percentage ranged from 0.19 to 0.38 [14–17]. In rainbow trout, a heritability estimate of 0.25–0.40 for muscle fat content has been reported [18,19]. Studies on QTL mapping for fat content were reported in only a few fish species. For instance, a significant QTL associated with fat percentage was detected on linkage group LNS16 in Atlantic salmon, and a marker (Ssa0016NVH) tightly linked to the QTL was found [20]. In common carp, a primary whole genome scan for QTL affecting flesh fat content was carried out in 8 full-sib families including 522 offspring [21]. In the study, evidence of a significant QTL was identified on LG13, accounting for 36.2% of the phenotypic variance. In addition, three suggestive QTLs with smaller effects were also detected. However, genome-wide association studies on fat content in aquatic species have just begun. So far, only one GWAS of fat content in Atlantic salmon has been reported, whereby 5 SNPs with significant test scores were identified on chromosomes 9 and 10 [22]. Besides, QTL for traits related to meat quality in fish, e.g. flesh color [23], muscle fiber [24], and omega-3 fatty acid [25], have been published in recent years.

From the above, the genetic architecture of fish flesh quality trait is still unclear. In the past ten years, GWAS has proven to be a powerful tool for identifying genetic variants associated with complex diseases and traits [26]. Many studies have been conducted to explore the genetic mechanism of various complex diseases and economic traits [27]. The recent development of high-throughput SNP genotyping array [8] facilitates GWAS analysis and identification of linked SNPs affecting phenotypic traits of interest in this species. In the present study, a GWAS was conducted for muscle fat content and abdominal traits in common carp derived from an F_2_ family using the high-density SNP chips (250 K) to detect potential SNPs and candidate genes. These results will eventually help develop breeding strategies for improving flesh quality in common carp.

## Materials and Methods

### Ethics Statement

This study was approved by the Animal Care and Use Committee of Heilongjiang River Fisheries Research Institute of Chinese Academy of Fishery Sciences. The methods were taken in accordance with approved guidelines. Before the muscle and blood samples were collected, all fishes were euthanized by being immersed in MS222 solution.

### Source of fish and phenotypic measurements

A full-sib F_2_ family containing 220 individuals was used in this study. This family was generated by intercrossing two F_1_ parents originated from a mass cross among 60 brooders, including 40 female and 20 male fishes. All fishes were cultured in the Hulan Aquaculture Experimental Station of Heilongjiang River Fisheries Research Institute, Harbin, China. After hatching, groups of fry were selected for rearing in a pond for 6 months. A total of 220 progenies were randomly collected and used for genotyping. After anesthesia with MS222, growth related traits were measured and blood samples (200–500μl) were collected from the caudal peduncle. The removable adipose tissues depositing in abdominal cavity and surrounding the viscera were weighed as AbFW. Then eviscerated weight (EW) was weighed, and the percentage of AbFW to EW was calculated as AbFP. Dorsal and abdominal muscle samples (~50 g) from each fish were taken and kept at -80°C for DNA extraction and analysis of fat content, respectively. Total lipids were extracted from the dorsal (MFdo) and abdominal (MFab) muscles of each fish and determined in duplicate according to Folch *et al*. [28]. The mean results from the two samples were used in the statistical analyses.

### Genotyping and quality control

Genomic DNA was isolated from whole blood using a QIAamp DNA Blood Mini Kit (Qiagen, Crawley, UK) following manufacturer's protocol. DNA was quantified using spectroscopy by Nanodrop 8000 (Thermo Scientific), and the integrity of DNA was checked by 1.5% agarose gel electrophoresis stained with ethidium bromide. DNA concentration was diluted to 50 ng/ul and the quality of genomic DNA met the requirement for the Illumina Infinium SNP genotyping platform.

We have developed a common carp 250 K SNP array using Affymetrix Axiom genotyping technology [8]. Genotyping using the common carp 250 K SNP array was outsourced to GeneSeek (Lincoln, Nebraska, USA). Genotypic data is available on all 220 F_2_ offspring as well as their parents and grandparents.

Quality control was carried out using PLINK v1.9 (https://www.cog-genomics.org/plink2) [29]. Samples with a < 95% call rate were excluded and 210 individuals were finally used for statistical analysis. SNPs were removed if they had genotype rate that is < 95% or a minor allele frequency (MAF) that is < 5%. Finally, Mendelian inheritance errors also were checked.

### Association analysis

The descriptive statistics of the traits were completed using SPSS 13.0. All traits (MFdo, MFab, AbFW and AbFP) deviated from normality, and Box-Cox transformation was applied using R software (http://www.r-project.org/).

The GWAS analysis for four traits used the univariate linear mixed model implemented in genome-wide efficient mixed-model association (GEMMA) [30]. To improve power to detect associations, we imputed all missing genotypes using beagle genetic analysis software (version 4.0) [31] before association testing. The GWAS was performed using the options to create a centered relatedness matrix (–gk 1) and perform all three frequentist analysis tests (-lmm 4): Wald, linklihood ratio and score. A covariate file (-c) including batch was incorporated into the mixed model.

The genome-wide significant/suggestive threshold *P*-value was decided based on Bonferroni-correction, we used the simpleM method [32] to correct the number of multiple tests. With this approach, an effective number of 8,992 independent SNPs was suggested; hence, the genome-wide significant and suggestive *P*-values were 0.05/8,992 = 5.56×10^−6^ (−log10 (*P*-value) = 5.25) and 1/8,992 = 1.11×10^−4^ (−log10 (*P*-value) = 3.95), respectively.

The Manhattan plot and Quantile-quantile (Q-Q) plots were made by qqman package available on the Comprehensive R Archive Network (http://cran.r-project.org/package=qqman).

### Annotation of Candidate Genes

The sequences of SNPs associated with muscle fat content and abdominal fat traits were compared by BLAST against the first version assembly of the common carp genome sequencing project [9], which is publicly available on www.carpbase.org.

### Quantitative measurement of mRNA

Possible candidate genes for MFdo, identified by the GWAS, were assessed in the dorsal muscle tissues using real-time quantitative PCR (Q-PCR). Tissue samples selected from individuals at the extremes of the phenotypic rankings as high (n = 10) and low (n = 10), as MFdo (high = 9.56 ± 0.72, low = 1.89 ± 0.25). Wu’s methods [33] were referred to for the methods of Q-PCR. Primers for the genes were designed from primer 3.0 (http://primer3.ut.ee) (S1 Table).

## Results

### Phenotype statistics

The descriptive statistics of the phenotypic measurements of muscle fat content and abdominal fat traits used for the present GWAS studies are given in Table 1. Means for MFdo, MFab, AbFW and AbFP were 4.89%, 7.79%, 7.12g and 1.89%, respectively. All non-normal phenotypic data were normalized after the Box-Cox transformation (S2 Table).

**Table 1:** Descriptive statistics of phenotypic data.

Traits (Unit)	N	Min	Max	Mean	SD	CV
MFdo (%)	215	1.5	11.2	4.89	1.73	35.38
MFab (%)	215	1.9	16.2	7.79	2.86	36.71
AbFW (g)	213	2.6	20.1	7.12	3.46	48.60
AbFP (%)	208	0.58	4.55	1.89	0.69	36.51

MFdo, fat content in dorsal muscle; MFab, fat content in abdominal muscle; AbFW, abdominal fat weight; AbFP, percentage of AbFW to eviscerated weight; N, number of individual; Min, minimum; Max, maximum; SD, standard error; CV, coefficient of variation. The same abbreviations are used in the following table.

### Genotyping and quality control

We assessed the SNP quality and excluded 56,869 SNPs with a call rate that is < 95%, 105,737 SNPs with MAF that is < 5%, and 1,576 SNPs with non-Mendelian segregation patterns. The SNP located on 50 chromosomes and those not assigned in the chromosomes and linkage maps were also excluded. Therefore, a set of 85,818 SNPs was included for GWAS (S3 Table). The distribution of SNPs after quality control and marker density on each chromosome is shown in S4 Table.

### Association analysis

**Fig 1:**
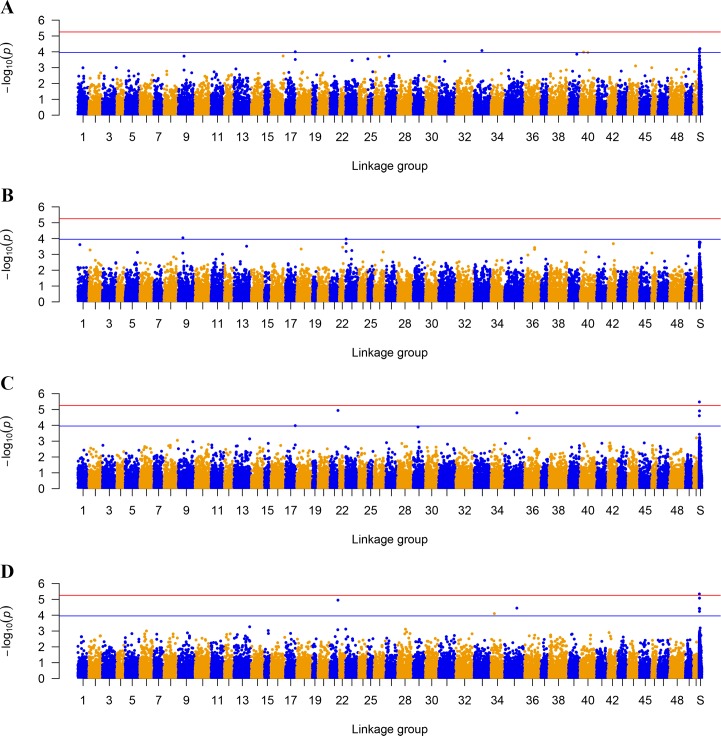
Manhattan plots of genome-wide association results. The red and blue solid line indicates the threshold *P*-value for genome-wide significant (5.56E-06) and suggestive (1.11E-04) level, respectively. Abbreviations: S, scaffold; A, fat content in dorsal muscle (MFdo); B, fat content in abdominal muscle (MFab); C, abdominal fat weight (AbFW); D, percentage of AbFW to eviscerated weight (AbFP).

We identified a total of 18 SNPs that achieved the suggestive significance (*P* < 1.11× 10^−4^) level for the four traits. Only one SNP (carp089419) surpassed the genome-wide significance (*P* < 5.56 × 10^−6^) level for both AbFW and AbFP (Fig 1 and Table 2).

**Table 2:** SNPs surpassing the suggestive significance level for muscle fat content and abdominal fat traits.

Trait	LG/Scaffold^a^	SNP	Position (bp)	Min/Maj^b^	MAF^c^	GWAS*P*-value^d^	Nearest Gene^e^	Location^f^
MFdo	17	carp139438	14592774	C/T	0.321	9.96E-05	*ankrd10a*	CDS
	33	carp192675	11483577	C/A	0.307	8.34E-05		Intergenic
	40	carp196436	5193668	A/G	0.314	1.05E-04	*tanc2*	CDS
	40	carp160064	11580617	A/C	0.304	1.11E-04	*tanc2*	CDS
	000000633	carp151503	840247	G/A	0.319	6.27E-05	*fjx1*	CDS
	000000761	carp100237	475274	T/C	0.295	1.07E-04		Intergenic
	000001231	carp249684	149043	T/A	0.309	1.09E-04	*chka*	U
	000002508	carp040917	29605	A/G	0.295	7.29E-05	*adam8a*	CDS
MFab	9	carp077510	7112924	A/G	0.490	9.02E-05	*zfand5a*	CDS
	23	carp153837	560892	A/G	0.483	1.07E-04		Intergenic
AbFW	17	carp139438	14592774	C/T	0.322	1.05E-04	*ankrd10a*	CDS
	21	carp244344	11825770	C/T	0.393	1.15E-05	*iffo2*	U
	35	carp202744	18344603	A/C	0.390	1.63E-05	*kpna4*	Intron
	000000247	carp144593	4006	C/T	0.388	2.48E-05		Intergenic
	000000386	carp089419	61091	T/C	0.395	**3.33E-06**		Intergenic
	000000695	carp128748	216424	A/G	0.393	1.23E-05	*clcn3*	Intron
AbFP	21	carp244344	11825770	C/T	0.390	1.12E-05	*iffo2*	U
	34	carp196106	4811371	T/C	0.497	7.95E-05	*apoer2*	D
	35	carp202744	18344603	A/C	0.388	3.57E-05	*kpna4*	Intron
	000000247	carp144593	4006	C/T	0.385	3.72E-05		Intergenic
	000000386	carp089419	61091	T/C	0.393	**4.56E-06**		Intergenic
	000000695	carp128746	215184	G/C	0.505	5.75E-05	*clcn3*	Intron
	000000695	carp128748	216424	A/G	0.390	8.35E-06	*clcn3*	Intron
	000028813	carp058937	486629	C/A	0.502	3.87E-05		Intergenic

^a ^SNP location on linkage group or scaffold in the common carp 250K array

^b^ Minor allele/Major allele

^c^ minor allele frequency

^d^ the bold *P*-values of SNPs are the genome-wide significant level

^e^ Gene location on the common carp assembly version1

^f^ D and U indicate that the SNP is downstream and upstream of the gene (< 5 Kb), respectively; CDS indicates coding sequence region.

The SNPs associated with muscle fat content and abdominal fat traits are presented in Table 2. We did not detect any genome-wide significant SNPs for muscle fat content. Of the 8 SNPs associated with MFdo, 4 were located on 3 LGs, while the remainders were mapped to scaffolds. Five SNPs were located in the known gene, of which two SNPs (carp196436 and carp160064) located within a 6.3Mb region on LG40 are both in the CDS of the tetratricopeptide repeat, ankyrin repeat and coiled-coil containing 2 (*tanc2*). Four SNPs (carp139438, carp151503, carp249684 and carp040917) were located within the genes for ankyrin repeat domain 10a (*ankrd10a*), four jointed box 1 (*fjx1*), choline kinase alpha (*chka*) and ADAM metallopeptidase domain 8a (*adam8a*), respectively. For MFab, 2 suggestive SNPs were identified on LGs. The SNP (carp077510), located at 7.11 Mb on LG9, was identified in the zinc finger AN1-type domain 5a (*zfand5a*) gene. The other SNP (carp153837) was located at 0.56Mb on LG23.

One genome-wide significance and 5 genome-wide suggestive significance SNPs were related to AbFW. Four SNPs were located within known genes. Notably, carp139438, locating in the *ankrd10a* gene, was also associated with MFdo. Three SNPs (carp244344, carp202744 and carp128748) were located within the genes for intermediate filament family orphan 2 (*iffo2*), karyopherin subunit alpha 4 (*kpna4*) and chloride channel 3 (*clcn3*), respectively. Eight loci associated with AbFP were identified on 3 LGs and 5 scaffolds. Only carp089419 surpassed the genome-wide significance level, which is also related to AbFW. Besides, four SNPs (carp244344, carp202744, carp144593 and carp128748) were found to be associated with both AbFW and AbFP. Two SNPs (carp128746 and carp128748), harbored within a 1.24 Kb region on scaffold000000695, were both identified in the intron of the *clcn3* gene. In addition, carp196106, located at 4.81 Mb on LG34, which is harbored in the downstream of apolipoprotein E receptor 2 (*apoer2*).

### The mRNA expression study of candidate genes

**Fig 2:**
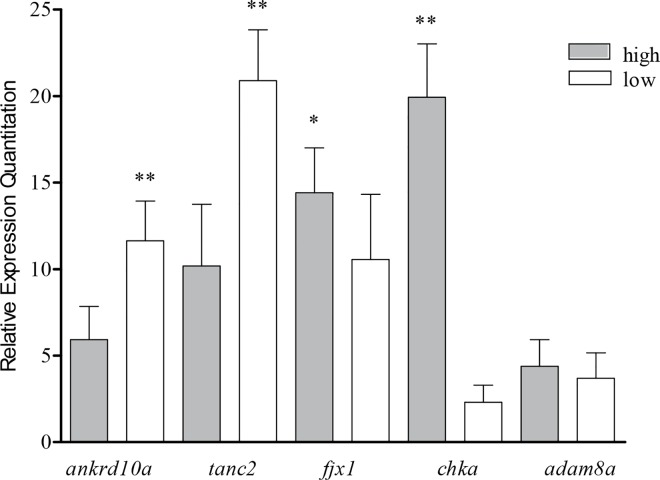
Relative mRNA abundance in the high and low dorsal muscle fat content groups. Relative expression levels were obtained by the 2^-ΔΔCT^ method; *18S* was used as normalizing controls. High group (n = 10) consisting of samples from common carp with the highest trait values and Low group (n = 10) with the lowest trait values; *, *P* < 0.05; ** *P* < 0.001.

Based on the GWAS analysis, 5 genes (*ankrd10a*, *tanc2*, *fjx1*, *chka* and *adam8a*) containing or in proximity to SNPs associated with MFdo were further validated by Q-PCR in subsets of eight individuals with lowest or highest trait phenotypic values. Significant differential expression (*P* < 0.05 or <0.001) between the low and high individuals was showed for 4 (*ankrd10a*, *tanc2*, *fjx1* and *chka*) of the 5 (Fig 2).

## Discussion

GWAS have been offering increased opportunities for detecting susceptibility loci for complex traits. Many published GWAS with high-density SNP markers are based on study designs with the samples of unrelated individuals [27]. It is generally accepted that association analysis using unrelated individuals is more powerful than using related individuals. However, family-based designs have several advantages, including quality control, well-known robustness to population stratification [34] and the ability to offer a variety of genetic analyses that cannot be achieved by using a sample of unrelated individuals, such as testing of parent-of-origin effects [35], detecting Mendelian errors, testing whether a genetic variant is inherited or *de novo* and combined linkage and association analysis [36]. Recently, a large number of family-based GWAS have been conducted on livestock [37]. Here, we present a GWAS of muscle fat content and abdominal fat traits in an F_2_ resource population of common carp. One significant (*P* < 5.56 × 10^−6^) and 17 suggestive SNPs (*P* < 1.11× 10^−4^) associated with 4 traits (MFdo, MFab, AbFW and AbFP) were identified. Moreover, with the aid of the common carp genome and gene annotation information, 10 possible candidate genes were screened. However, the sample size was still relatively limited in the current study, which may be the reason why genome-wide significance was not achieved on MFdo and MFab. In addition, a larger number of SNPs may be from genes that have not been annotated, or located on scaffolds. Therefore, further annotations on current common carp assembly version are necessary.

Muscle fat is an important determinative factor of flesh quality influencing the juiciness, drop, texture and flavor of meat [12]. As revealed from this study, 5 genes (*ankrd10a*, *tanc2*, *fjx1*, *chka* and *adam8a*) are shown to be potentially associated with MFdo, and they were selected for measurement of mRNA expression. Four (*ankrd10a*, *tanc2*, *fjx1* and *chka*) of 5 genes were differentially expressed in individuals with high and low MFdo (Fig 2), some are known to play roles in lipid metabolism. The gene *chka* plays an important role in lipid, lipoprotein and choline metabolism, which is involved in very low-density lipoprotein secretion, making this nutritional pathway an important contributor to hepatic lipid balance [38]. This gene was differentially expressed in the duodenum tissue between the low and high residual feed intake chickens [39]. In addition, the *chka* gene encodes a protein essential in the phospholipid biosynthesis and may contribute to human carcinogenesis [40]. Polymorphisms in *chka* have been identified to be associated with human diabetes [41]. The *tanc2* gene has been investigated as a candidate gene for the locus on BAT19 where BTB-00755526 at 48.24 Mb was associated with mirystic acid (C14:0) of intramuscular adipose tissue in Japanese Black cattle [42]. Interestingly, this gene was up-regulated in the high-fat groups compared to the low-fat groups in pig longissimus muscle at 110 kg Body Weight [43]. Two SNPs were detected here as being associated with MFab, while only one annotated genes (*zfand5*) were identified. The *zfand5* was down-regulated in the obese group compared with the lean group of dogs [44].

In addition to muscle fat content, other fat traits, and especially abdominal fat, are important selection goal in animal breeding, e.g. chicken [45], and pig [46]. In the present study, 4 genes (*ankrd10a*, *iffo2*, *kpna4* and *clcn3*) containing or near SNPs associated with AbFW were identified. Three genes, *iffo2*, *kpna4* and *clcn3*, were found to be associated with both AbFW and AbFP. In addition, *apoer2* was also related to AbFP. The gene *ankrd10a* has been detected as a candidate gene for abdominal fat yield on GGA3 in chicken [47]. The gene *iffo2* has been associated with residual body weight gain in cattle [48]. The *apoer2* (also known as low-density lipoprotein receptor-related protein 8, *lrp8*) is a member of the low density lipoprotein receptor (LDLR) gene family, which is not only involved in the lipid metabolism, but also plays an important role in endocytosis and signal transduction [49]. Polymorphisms in the *lrp8* gene can influence plasma cholesterol levels as well as the size and composition of LDL particles [50]. Furthermore, *lrp8* is also involved in other physiologically processes, e.g. lipid-raft sorting, endothelial cell migration and fetal growth [51,52]. In addition, Yao *et al*. reported that *lrp8* gene was a new member of eggshell matrix protein and significantly associated with eggshell traits [53].

## Conclusion

To our knowledge, this might be the earliest GWAS study on flesh quality traits in common carp. In summary, one genome-wide significant SNP and 17 suggestive SNPs for 4 traits were identified in a F_2_ common carp population. Among them, one SNP (carp139438), and 4 SNPs (carp244344, carp202744, carp144593 and carp128748) for AbFW were also found to be involved in MFdo and AbFP, respectively, indicating a pleiotropic effect. Based on the association signals and differential expression of harbored genes, 4 candidate genes for MFdo were identified. The results would contribute to further exploration into the potential mechanism of flesh quality traits and improve our understanding of the biology of fat metabolism and deposition in common carp.

## Supporting Information

S1 Table Primers used for Q-PCR validation. (DOCX)

S2 Table Descriptive statistics of phenotypic data after the transformation. (DOCX)

S3 Table Genotypes of all individuals after quality control. (ZIP)

S4 Table Distribution of SNP markers after quality control and marker density on each linkage group. (DOCX)

S1 Fig Quantile-quantile (Q-Q) plots of genome-wide association results. MFdo, fat content in dorsal muscle; MFab, fat content in abdominal muscle; AbFW, abdominal fat weight; AbFP, percentage of AbFW to eviscerated weight.
(TIF)
