# Supplementary material for: Genome-Wide Association Study for Muscle Fat Content and Abdominal Fat Traits in Common Carp (Cyprinus carpio)
Source: PLoS One. 2016 Dec 28;11(12):e0169127. doi: 10.1371/journal.pone.0169127 (PMC5193488; doi:10.1371/journal.pone.0169127)
Supplement: S1 Table — (DOCX) [file pone.0169127.s001.docx]

S1 Table. Primers used for Q-PCR validation

| Gene name | Primer sequence (5’-3’) | Production length (bp) | Tm (℃)^1^ |
| --- | --- | --- | --- |
| *ankrd10a* | F: TCCAAAAACTGCTGCCACAC  R: ACCTCGACAGATTGCCAGTG | 100 | 60 |
| *tanc2* | F: TTTTCCCCGAAGCTGACCAC  R: GGCGTCACACACTACCGAAA | 284 | 61 |
| *fjx1* | F: AGGAAGTGGAGAGAGCAGGT  R: TTCTCCCAGCACTTGATCCG | 147 | 60 |
| *chka* | F: AGAAGAAATGGGAGGCCAGC  R: CACCAAAAACTACCCCACCAAATC | 164 | 60 |
| *adam8a* | F: TGGATTCCTCGCCCACAGAT  R: TACACACCCCACGACTGTTG | 150 | 60 |
| *18S*^2^ | F: ACGATCAGATACCGTCGTAGTTCC  R: CTGTCAATCCTTTCCGTGTCCG | 244 | 60 |

^1^ The annealing temperature represents the optimal temperature during quantitative PCR; ^2^ mRNA levels of *18S* was assayed for normalization during quantitative PCR.
