# Supplementary material for: Genome-Wide Association Study for Muscle Fat Content and Abdominal Fat Traits in Common Carp (Cyprinus carpio)
Source: PLoS One. 2016 Dec 28;11(12):e0169127. doi: 10.1371/journal.pone.0169127 (PMC5193488; doi:10.1371/journal.pone.0169127)
Supplement: S2 Table — (DOCX) [file pone.0169127.s002.docx]

S2 Table. Descriptive statistics of phenotypic data after the transformation

| Trait | Min | Max | Mean | SD | λ |
| --- | --- | --- | --- | --- | --- |
| MFdo | 0.439 | 3.973 | 2.082 | 0.786 | 0.3852 |
| MFab | 0.843 | 10.629 | 5.266 | 1.984 | 0.8147 |
| AbFW | 0.801 | 1.788 | 1.315 | 0.217 | -0.3811 |
| AbFP | -0.591 | 1.224 | 0.522 | 0.282 | -0.2924 |

MFdo, fat content in dorsal muscle; MFab, fat content in abdominal muscle; AbFW, abdominal fat weight; AbFP, percentage of AbFW to eviscerated weight; Min, minimum; Max, maximum; SD, standard error; CV, λ, parameter transformation.
