# Supplementary material for: Genome-Wide Association Study for Muscle Fat Content and Abdominal Fat Traits in Common Carp (Cyprinus carpio)
Source: PLoS One. 2016 Dec 28;11(12):e0169127. doi: 10.1371/journal.pone.0169127 (PMC5193488; doi:10.1371/journal.pone.0169127)
Supplement: S4 Table — (DOCX) [file pone.0169127.s004.docx]

S4 Table. Distribution of SNP markers after quality control and marker density on each linkage group

| LG^1^ | NO. of  SNPs | Density  (Kb/SNP) | LG | NO. of  SNPs | Density  (Kb/SNP) |
| --- | --- | --- | --- | --- | --- |
| 1 | 800 | 19.03 | 27 | 740 | 21.98 |
| 2 | 1032 | 18.32 | 28 | 1273 | 17.19 |
| 3 | 982 | 20.83 | 29 | 892 | 19.43 |
| 4 | 594 | 19.02 | 30 | 945 | 19.39 |
| 5 | 1281 | 16.31 | 31 | 1480 | 16.53 |
| 6 | 1077 | 18.21 | 32 | 1340 | 19.44 |
| 7 | 767 | 18.41 | 33 | 1263 | 18.99 |
| 8 | 1069 | 18.49 | 34 | 968 | 19.36 |
| 9 | 1292 | 18.65 | 35 | 1339 | 21.61 |
| 10 | 1267 | 17.78 | 36 | 1203 | 18.87 |
| 11 | 1094 | 17.27 | 37 | 663 | 16.82 |
| 12 | 713 | 17.80 | 38 | 1647 | 16.86 |
| 13 | 1256 | 19.82 | 39 | 814 | 19.74 |
| 14 | 871 | 21.00 | 40 | 1173 | 19.92 |
| 15 | 413 | 20.32 | 41 | 761 | 20.46 |
| 16 | 975 | 20.92 | 42 | 810 | 16.71 |
| 17 | 782 | 20.02 | 43 | 723 | 19.58 |
| 18 | 1080 | 20.63 | 44 | 852 | 18.54 |
| 19 | 429 | 16.80 | 45 | 803 | 21.39 |
| 20 | 814 | 20.40 | 46 | 338 | 22.82 |
| 21 | 686 | 18.82 | 47 | 701 | 23.53 |
| 22 | 444 | 18.65 | 48 | 1093 | 20.52 |
| 23 | 938 | 19.61 | 49 | 612 | 17.98 |
| 24 | 681 | 18.11 | 50 | 365 | 20.59 |
| 25 | 459 | 18.52 | Scaffold^2^ | 40464 |  |
| 26 | 760 | 21.45 | Total | 85818 |  |

^1^ Linkage group; ^2^ These SNPs are not assigned to any linkage groups.
