# Supplementary figures and images for: Genome-Wide Association Study for Muscle Fat Content and Abdominal Fat Traits in Common Carp (Cyprinus carpio)
Source: PLoS One. 2016 Dec 28;11(12):e0169127. doi: 10.1371/journal.pone.0169127 (PMC5193488; doi:10.1371/journal.pone.0169127)

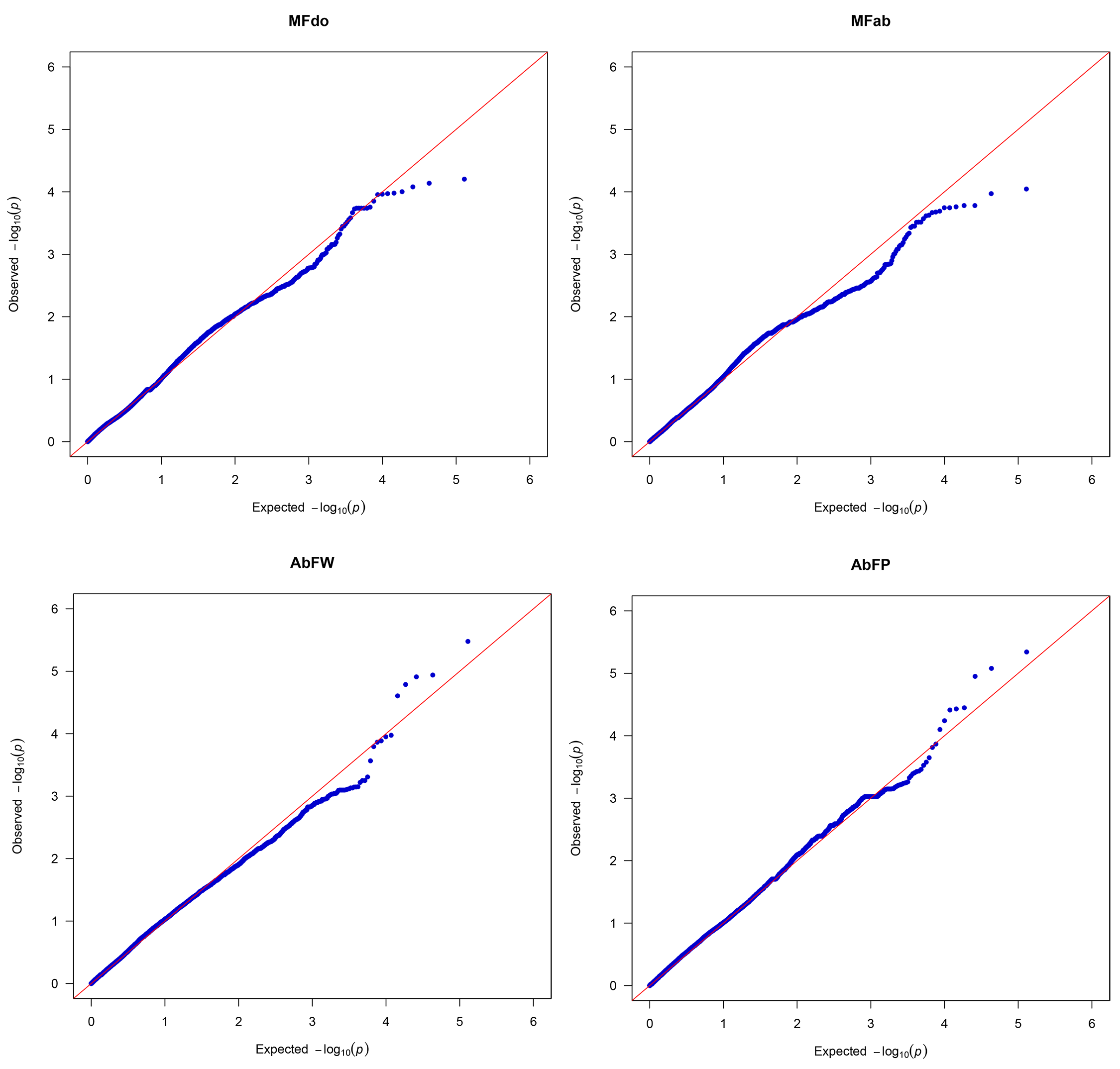

Supplement: S1 Fig — MFdo, fat content in dorsal muscle; MFab, fat content in abdominal muscle; AbFW, abdominal fat weight; AbFP, percentage of AbFW to eviscerated weight. (TIF) [file pone.0169127.s005.tif]
